# Luteolin as a dietary flavonoid for brain health: modulating neuroinflammation and cognitive decline in neurodegenerative disorders

**DOI:** 10.3389/fnut.2026.1774416

**Published:** 2026-04-07

**Authors:** Huanglei Jiang, Xiu'e Pang

**Affiliations:** 1 Chinese Medicine Hospital of Tiantai County, Taizhou, Zhejiang, China; 2 Department of Neurology, Tiantai People's Hospital of Zhejiang Province, Tiantai Branch of Zhejiang Provincial People's Hospital, Hangzhou Medical College, Taizhou, Zhejiang, China

**Keywords:** cognitive decline, luteolin, neurodegenerative diseases, neuroinflammation, oxidative stress

## Abstract

Luteolin, a flavonoid naturally present in a variety of fruits, vegetables, and medicinal plants, has been recognized as a potentially effective neuroprotective nutraceutical because of its remarkable anti-inflammatory, antioxidant, and neurotrophic properties. Increasing evidence suggests that neuroinflammation and oxidative stress are major contributors to cognitive decline and neuronal degeneration in several prominent neurodegenerative disorders, including Alzheimer's disease (AD), Parkinson's disease (PD), Huntington's disease (HD), and multiple sclerosis (MS). Luteolin significantly inhibits microglial activation, reduces pro-inflammatory cytokine production, modulates the nuclear factor kappa B (NF-κB) and mitogen-activated protein kinase (MAPK) signaling pathways, and enhances Nrf2-mediated antioxidant mechanisms. Furthermore, it promotes synaptic plasticity through brain-derived neurotrophic factor (BDNF)-associated pathways and mitigates the aggregation of pathological proteins, including Aβ, tau, α-synuclein, and mutant huntingtin. Preclinical studies consistently demonstrate substantial improvements in cognitive function, motor performance, demyelination, and neuronal viability in models of AD, PD, MS, and HD. Preliminary clinical observations also indicate prospective advantages for cognitive function, regulation of inflammatory responses, and alleviation of symptoms, particularly concerning AD and MS. Notwithstanding these encouraging outcomes, obstacles persist due to luteolin's restricted bioavailability, ideal dosing parameters, and the translational discrepancies between experimental models and human pathophysiological conditions. In summary, luteolin emerges as a noteworthy candidate for nutraceutical-oriented approaches designed to alleviate neuroinflammation and cognitive deterioration in the context of neurodegenerative diseases.

## 1 Introduction

Neurodegenerative disorders (NDDs), including Alzheimer's disease (AD), Parkinson's disease (PD), Huntington's disease (HD), and amyotrophic lateral sclerosis (ALS), represent one of the most pressing global health challenges of the 21st century. As population aging accelerates worldwide, the incidence of NDDs has risen dramatically, with more than 55 million people currently living with dementia and projections suggesting that this number will exceed 139 million by 2050 (1). These conditions are characterized by progressive neuronal degeneration, accumulation of misfolded proteins, chronic neuroinflammation, and synaptic dysfunction, ultimately leading to cognitive decline, motor impairment, and loss of independence (2, 3). Despite considerable advances in understanding their molecular pathogenesis, current pharmacological treatments are largely symptomatic and do not alter disease progression, underscoring the urgent need for safe and effective preventive and therapeutic interventions.

In contemporary scientific discourse, nutraceuticals and bioactive dietary constituents have garnered heightened scrutiny as promising neuroprotective agents. These naturally occurring compounds demonstrate a spectrum of beneficial activities, including antioxidant, anti-inflammatory, anti-apoptotic, and anti-amyloidogenic properties, which specifically target the fundamental mechanisms that contribute to neurodegeneration (4, 5). In contrast to traditional pharmacological interventions, nutraceuticals present several advantages, including reduced toxicity, diverse biological effects, widespread availability, and a favorable safety profile over prolonged periods, thereby rendering them appealing candidates for both preventive measures and adjunctive therapeutic strategies (6, 7). Notably, among these bioactive compounds, flavonoids have been recognized as particularly effective modulators of neuronal signaling pathways and neuroinflammatory responses.

Luteolin 3′, 4′, 5, 7-tetrahydroxyflavone is a flavonoid that occurs naturally and is predominantly present in various food sources such as celery, parsley, chamomile, green pepper, and a range of medicinal botanical species. It exhibits strong antioxidant capabilities, significant anti-inflammatory effects through the inhibition of nuclear factor kappa B (NF-κB) and mitogen-activated protein kinase (MAPK) signaling pathways, and the capacity to influence microglial activation, synaptic functionality, and oxidative stress (8–10). These physiological activities establish luteolin as a promising nutraceutical candidate for modulating neuroinflammation and cognitive decline, two key features associated with neurodegenerative disorders (NDDs). Accumulating preclinical and nascent clinical evidence indicates that luteolin is able to cross the blood-brain barrier (BBB), reducing neuroimmune activation, promoting neuronal viability, and enhancing cognitive performance in models of AD, PD, and other NDDs (11–13).

This review aims to provide a clear and up-to-date overview of the molecular mechanisms by which luteolin exerts neuroprotective effects. In particular, it focuses on how luteolin modulates neuroinflammation and helps prevent cognitive decline. We summarize recent findings from mechanistic studies and review available translational and clinical evidence. In addition, we discuss the potential role of luteolin in precision and preventive neuromedicine. By integrating molecular, cellular, and clinical insights, this review highlights luteolin as a promising nutraceutical for the prevention and management of NDDs. It also identifies important knowledge gaps and suggests future research directions needed for clinical application.

## 2 Chemistry, sources, and pharmacokinetics of luteolin

### 2.1 Chemical structure and classification

Luteolin represents a naturally occurring flavone that is part of the broader class of polyphenolic flavonoids. From a structural perspective, luteolin is defined by the presence of two benzene rings (A and B) interconnected by a heterocyclic pyrone ring (C), thereby establishing the characteristic C6–C3–C6 backbone that is emblematic of flavonoids (14). The existence of four hydroxyl groups situated at positions 3′, 4′, 5, and 7 significantly enhances its potent antioxidant properties, metal-chelating capacity, and abilities to neutralize free radicals (15). Furthermore, its chemical structure plays a critical role in modulating cellular signaling pathways, particularly those involved in oxidative stress and inflammatory responses. Luteolin is classified within the flavone subclass, a category recognized for its distinctive double bond between C2–C3 and a carbonyl group at C4, characteristics that augment lipophilicity and promote interactions with cellular enzymes and receptors (14).

### 2.2 Natural dietary sources

Luteolin is extensively found in traditional herbal remedies and nutritional supplements. Predominant sources within the diet encompass celery, parsley, thyme, oregano, chamomile, carrots, peppers, broccoli, and olive oil (16, 17). Substantial concentrations are also present in traditional herbal remedies such as Chrysanthemum morifolium, Perilla frutescens, and Sophora japonica commonly utilized in traditional practices for their anti-inflammatory properties (18). Additionally, tea, fruits including apples and oranges, and whole grains provide further contributions to the human dietary intake (19). The presence of luteolin in commonly consumed vegetables and herbs suggests that it may serve as a potentially accessible nutraceutical, although its actual dietary availability may vary and warrants further quantitative evaluation.

### 2.3 Absorption, metabolism, and bioavailability

Although luteolin demonstrates diverse biological activities and significant pharmacological potential, its oral bioavailability remains rather modest. The small intestine serves as the primary site of absorption, where luteolin appears either in its unbound aglycone state or linked to glycosides. The glycosylated forms of luteolin are broken down by intestinal β-glucosidases prior to absorption; nevertheless, some glycosides can pass through intact via the sodium-dependent glucose transporter SGLT1 (20). Following absorption, luteolin experiences a significant phase II metabolism within the intestinal mucosa and liver, leading to the formation of glucuronidated, sulfated, and methylated metabolites (20). These conjugated forms are the main constituents circulating in the bloodstream. The absolute oral bioavailability of luteolin is quite low due to its limited solubility and rapid metabolism; yet, its metabolites preserve considerable biological activity that may enhance its neuroprotective properties (21). Innovative approaches such as nano-formulations, phospholipid complexes, and the co-administration with bioenhancers have demonstrated the ability to elevate pharmacokinetic characteristics of luteolin (22).

### 2.4 BBB penetration

**Figure 1:**
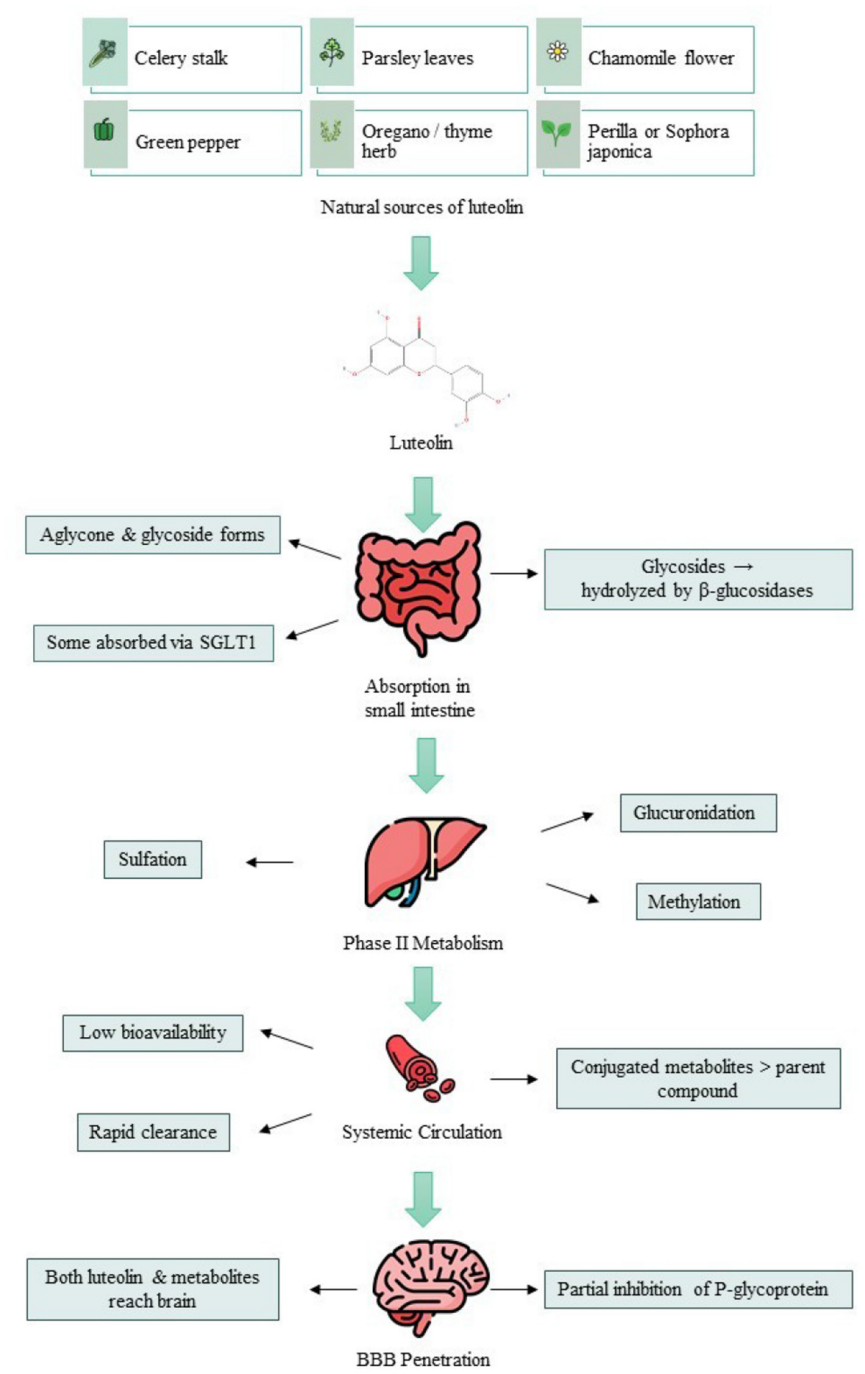
Luteolin Journey: from dietary sources to CNS effects. This diagram illustrates the main steps in luteolin's journey from food intake to systemic circulation. It highlights its natural sources, intestinal absorption, phase II metabolism, and subsequent transport in the bloodstream, outlining the key determinants shaping its overall bioavailability. This figure has been designed using resources from https://www.Flaticon.com.

Effective neuroprotection hinges upon the passage of bioactive compounds across the BBB. Preclinical investigations reveal that both free luteolin and its derivatives can amass in cerebral tissue subsequent to oral or intravenous administration (23). Luteolin's ability to inhibit efflux transporters such as P-glycoprotein may further enhance its penetration into the central nervous system (As illustrated in Figure 1, luteolin derived from dietary sources such as celery, parsley, chamomile, and green pepper undergoes intestinal absorption and phase II metabolism, enters systemic circulation, crosses the blood–brain barrier, and accumulates in the brain, where it exerts anti-inflammatory and antioxidant effects that contribute to neuroprotection) (13). Once nestled within the CNS, luteolin unleashes formidable anti-inflammatory and antioxidant effects, addressing microglial activation, cytokine release, and oxidative pathways pertinent to NDDs (24, 25).

### 2.5 Pharmacokinetics and safety

Pharmacokinetic investigations reveal that luteolin undergoes swift absorption; however, it experiences considerable first-pass metabolism, culminating in a brief plasma half-life of approximately 1–3 h, contingent upon the species and formulation utilized (20). The elimination of luteolin metabolites primarily occurs via renal excretion and enterohepatic circulation. Regarding safety, luteolin is regarded as well tolerated at physiologically pertinent dietary levels. Toxicological assessments have indicated an absence of genotoxicity or carcinogenicity, and high-dose studies conducted on animals reveal a substantial safety margin (26). Nonetheless, very high doses may demonstrate estrogenic or pro-oxidant properties, necessitating meticulous dose optimization for therapeutic applications. Investigations involving human subjects indicate satisfactory tolerability, with mild gastrointestinal discomfort identified as the most frequently reported adverse effect (26). Collectively, luteolin exhibits a commendable safety profile, bolstering its potential as a promising neuroprotective nutraceutical.

## 3 Mechanistic basis of luteolin's neuroprotective activity

Luteolin exerts neuroprotection through pleiotropic actions on inflammation, oxidative stress, synaptic function, cell death pathways and proteostasis (27, 28). The following sub-sections summarize major mechanistic axes, drawing on cellular, animal, and translational studies and on recent reviews that integrate these findings.

### 3.1 Anti-inflammatory mechanisms

Neuroinflammation, orchestrated primarily through the activation of microglia and astrocytes, acts as a trigger for the progression of neurodegenerative diseases. Luteolin exerts a substantial inhibitory effect on inflammatory signaling by disrupting critical transcriptional and kinase pathways. At the mechanistic level, luteolin obstructs the activation and nuclear translocation of NF-κB, consequently diminishing the transcriptional levels of pro-inflammatory cytokines and enzymes. Simultaneously, luteolin reduces signaling through the MAPK family, thereby attenuating downstream pro-inflammatory and pro-apoptotic responses (29, 30). Within microglial cells, luteolin inhibits classical (M1-like) activation, curtails the production of nitric oxide and reactive oxygen species (ROS), and promotes a phenotypic transition of microglia toward a more quiescent or reparative state, these actions mitigate collateral neuronal damage in models of AD, PD, and other neuroinflammatory disorders (4, 31). Collectively, these effects not only restrain acute inflammatory injury but also disrupt chronic feed-forward mechanisms that exacerbate neurodegeneration.

### 3.2 Antioxidant actions

Oxidative stress and mitochondrial dysfunction are hallmarks of neurodegenerative diseases. Luteolin serves as a direct scavenger of radicals due to its catechol-like pattern of hydroxylation and also activates the body's natural antioxidant defenses. Numerous investigations reveal that luteolin stimulates the Nrf2/ARE pathway, enhancing the production of phase II antioxidant enzymes and thus fortifying cellular endurance against ROS (10, 14). At the mitochondrial level, luteolin protects membrane potential, decreases ROS generation, and limits permeability transition; these actions collectively support ATP synthesis and prevent the release of pro-apoptotic elements (31, 32). By merging direct neutralization of free radicals with the transcriptional enhancement of protective cellular systems, luteolin mitigates oxidative harm to lipids, proteins, and DNA in neuronal paradigms.

### 3.3 Synaptic plasticity and neurotrophic support

The safeguarding of synaptic integrity and its remarkable adaptability is vital for our cognitive prowess. Early-stage research suggests that luteolin may elevate the levels of brain-derived neurotrophic factor (BDNF) and amplify the downstream tropomyosin receptor kinase B signaling, which is instrumental in nurturing dendritic spine upkeep, promoting synaptic protein expression, and facilitating processes tied to long-term potentiation (4, 33, 34). By weaving together its anti-inflammatory and antioxidant prowess, luteolin also plays a crucial role in shielding synaptic functionality by diminishing the synaptotoxicity wrought by cytokines and ROS (35, 36). These nourishing and protective actions converge to enhance learning and memory across various animal models exhibiting cognitive deficits.

### 3.4 Anti-apoptotic and anti-ferroptotic effects

Luteolin exerts neuroprotective effects against programmed cell death by modulating both intrinsic and extrinsic pathways; specifically, it inhibits pro-apoptotic mediators such as Bax and cleaved caspase-3 while simultaneously upregulating anti-apoptotic proteins like Bcl-2, thereby stabilizing mitochondrial integrity and obstructing cytochrome-c–dependent cascades (10). Recent investigations have also identified luteolin's role in mitigating ferroptosis, a regulated, iron-dependent form of lipid peroxidation that is associated with neurodegenerative processes. By diminishing lipid ROS, maintaining the activity of the glutathione/glutathione peroxidase 4 (GPX4) axis, and chelating labile iron indirectly through its antioxidant and metabolic properties, luteolin can attenuate the susceptibility to ferroptotic cell death in experimental models characterized by significant iron-driven oxidative damage (37, 38). Although there is a relative scarcity of ferroptosis-specific studies involving luteolin when compared to classical apoptosis, the compound's synergistic effects on redox balance, iron homeostasis, and mitochondrial functionality render its anti-ferroptotic activity a plausible and therapeutically significant mechanism.

### 3.5 Effects on protein aggregation

Luteolin exerts its influence on proteostasis through various mechanisms: it has the capacity to diminish the production and aggregation propensity of misfolded proteins indirectly by attenuating oxidative stress and neuroinflammation that promote misfolding, as well as by modulating proteolytic systems (4, 10). In models of AD, treatment with luteolin has been correlated with a decrease in Aβ accumulation and a reduction in tau hyperphosphorylation (39, 40). Concerning α-synuclein and huntingtin, the existing evidence is more constrained yet remains supportive: by enhancing cellular clearance pathways and mitigating oxidative environments conducive to oligomerization, luteolin may facilitate a reduction in the formation of toxic aggregates and the concomitant cellular stress (39, 40). Collectively, luteolin's intricate modulation of the cellular milieu plays a significant role in diminishing protein aggregation and toxicity across various neurodegenerative models.

## 4 Evidence from preclinical and clinical studies

### 4.1 AD

AD continues to be the predominant etiology of dementia, with existing therapeutic interventions providing merely marginal symptomatic alleviation while failing to substantially modify the trajectory of the disease (41). Consequently, the exploration of luteolin's involvement in AD is of paramount significance, as an increasing body of preclinical and clinical data suggests that this flavonoid addresses several pathological mechanisms integral to the progression of AD. Preclinical investigations consistently demonstrate that luteolin may mitigate amyloid-beta (Aβ)-induced neurotoxicity, inhibits tau hyperphosphorylation, modulates neuroinflammation driven by microglia, and enhances cognitive functions such as learning and memory in models of AD (11). These observations underscore its potential capacity to safeguard neuronal integrity and attenuate cognitive deterioration.

Although clinical evidence remains somewhat sparse, it further emphasizes the critical importance of examining luteolin within the context of AD. Preliminary trials indicate enhancements in cognitive performance alongside reductions in inflammatory biomarkers among subjects administered luteolin-enriched formulations (11). Collectively, this comprehensive assemblage of evidence reinforces the relevance of luteolin as an auspicious nutraceutical candidate for addressing the intricate pathophysiology of AD and for fostering innovative therapeutic approaches.

An investigation explored the underlying mechanisms through which the combination of luteolin and exercise mitigates cognitive deficits in a mouse model of AD induced by Aβ1-42 (42). Untargeted metabolomic analyses indicated that luteolin+exercise influences the metabolism of purine, retinol, thiamine, histidine, and cysteine/methionine, thereby enhancing energy homeostasis. Furthermore, this combination reinstated critical autophagy proteins, and the inhibition of autophagy was found to obstruct its cognitive advantages, thereby underscoring autophagy as a pivotal mediator of the neuroprotective properties of luteolin+exercise in the context of AD (42) (Table 1).

**Table 1:** Summary of luteolin-based interventions and their mechanisms in AD models.

Type of intervention	Model	Main results	Mechanism of action	Reference
Luteolin + Exercise	Aβ1-42-oligomers-induced AD mice	Cognitive impairment ↓, autophagy proteins ↑, energy metabolism disorder ↓	Modulation of autophagy; regulated purine, retinol, thiamine, histidine, cysteine, and methionine metabolism	Tao et al. (42)
Ginkgo biloba leaf extract (GBE)	AD mice	Cognitive function ↑, neuronal morphology ↑, inflammatory factors ↓	Modulation of PI3K/AKT/NF-κB signaling pathway	Zhu et al. (43)
Luteolin	3 × Tg-AD mice and primary neurons	Memory/cognition ↑, neuronal apoptosis ↓, mitochondrial damage ↓	PPARγ-dependent; inhibited Aβ generation and oxidative stress	He et al. (40)
Luteolin + Exercise	Aβ1-42 oligomers-induced AD mice	Cognitive performance ↑, Aβ content ↓, astrocyte/microglia activation ↓, autophagy ↑	Decreased neuroinflammation; enhanced autophagy; combination more effective than monotherapy	Tao et al. (44)
Co-ultramicronized palmitoylethanolamide + Luteolin (co-ultra PEALut)	Astrocyte/OPC co-culture with Aβ1-42	Oligodendrocyte morphology ↑, astrocyte reactivity ↓, OPC maturation ↓ (restored)	PPAR-α-mediated; counteracted Aβ1-42-induced deficits	Facchinetti et al. (45)
Luteolin-loaded chitosan nanoparticles (intranasal, 50 mg/kg)	Sporadic AD mice (ICV-STZ, 3 mg/kg)	Cognitive function ↑, neuronal survival ↑, amyloid plaques ↓, Aβ aggregation ↓, hyperphosphorylated tau ↓, oxidative stress ↓, pro-inflammatory mediators ↓	Antioxidant, anti-inflammatory, anti-amyloidogenic pathways; brain-targeted nanodelivery	Abbas et al. (46)
Luteolin-loaded bilosomes (intranasal, 50 mg/kg)	Sporadic AD mice (ICV-STZ, 3 mg/kg)	Short-term/long-term memory ↑, oxidative stress ↓, pro-inflammatory mediators ↓, Aβ aggregation ↓, hyperphosphorylated tau ↓	Antioxidant, anti-inflammatory, anti-amyloidogenic; enhanced brain delivery via bilosomes	Elsheikh et al. (47)
DHA + Luteolin + Urolithin A (synergistic combination, *in vitro*)	Human neuroblastoma BE(2)-M17 cells, Aβ1-42-induced toxicity	Cell viability ↑, Aβ1-42 toxicity ↓	Synergistic neuroprotection; nutraceutical combination reduces Aβ-induced cytotoxicity	Jayatunga et al. (48)
Luteolin (80 mg/kg/day, i.c.v.)	AD mice (Aβ1-42 oligomers injected)	Cognitive impairment ↓, p-JNK ↓, p38 MAPK ↓, GFAP ↓, Iba-1 ↓, TNF-α ↓, IL-1β ↓, Bax ↓, Bcl-2 ↑, Caspase-3 ↓, Cox-2 ↓, BACE-1 ↓, Aβ1-42 ↓, PSD-95 ↑, SNAP-25 ↑	Inhibition of JNK pathway; reduces neuroinflammation, amyloidogenesis, apoptosis, and synaptic dysfunction	Ahmad et al. (49)
Luteolin (20, 40 mg/kg/day, i.p., 3 weeks)	3 × Tg-AD mice	Spatial learning ↑, memory deficits ↓, GFAP ↓, TNF-α ↓, IL-1β ↓, IL-6 ↓, NO ↓, COX-2 ↓, iNOS ↓, ER stress markers GRP78 ↓, IRE1α ↓	Inhibition of ER stress-dependent neuroinflammation; antioxidant and anti-inflammatory activity	Kou et al. (50)
*In silico* evaluation of flavonoids (luteolin, apigenin, ellagic acid, others) from medicinal plants	Computational models (AChE, BChE, MAO targets)	Predicted binding affinity ↑ (luteolin highest), toxicity ↔, absorption ↑	Pharmacophore modeling, Auto-QSAR, molecular docking; inhibition of AChE, BChE, MAO	Ojo et al. (51)
Luteolin (*in vitro*, dose not specified)	PC-12 cells, Aβ25-35-induced apoptosis	Cell viability ↑, apoptosis ↓, Bcl-2 ↑, Bax ↓, caspase-3 ↓, p-ERK1/2 ↑	Activation of ER/ERK/MAPK pathway via ERβ; anti-apoptotic effect	Wang et al. (52)
Luteolin + l-theanine (0.05% + 0.1% in diet, 8 weeks)	Rats, Aβ25-35 infusion into hippocampal CA1	Memory function ↑, hippocampal insulin signaling ↑, TNF-α ↓, norepinephrine degradation ↓, glucose infusion rates ↑, hepatic glucose output ↓	Potentiation of hippocampal insulin signaling (pAkt → pGSK → pTau), anti-inflammatory, improvement in norepinephrine metabolism	Park et al. (53)
Luteolin (5, 10, 15, 20 μM in diet)	Transgenic Drosophila expressing human Aβ42	Lifespan ↑, climbing ability ↑, activity ↑, LPO ↓, PCC ↓, GST ↓, GSH ↑, SOD ↓, CAT ↓, AChE ↓, caspase-3/9 ↓, Aβ42 plaque ↓	Anti-oxidative, anti-apoptotic, inhibition of AChE, prevention/disaggregation of Aβ42 plaques	Ali et al. (54)
Luteolin	Rats, STZ-induced AD	Spatial learning ↑, memory ↑, CA1 pyramidal layer thickness ↑	Neuroprotective effect on hippocampal structures, amelioration of STZ-induced neuronal damage	Wang et al. (55)
Co-ultramicronized Palmitoylethanolamide + Luteolin (co-ultraPEALut, 27, 2.7, 0.27 μM PEA)	*In vitro*: differentiated SH-SY5Y neurons; *ex vivo*: mouse hippocampal slice cultures	Astrocyte activation ↓, GFAP ↓, iNOS ↓, neuronal nitric oxide synthase ↑, BDNF ↑, apoptosis ↓	Anti-inflammatory and neuroprotective via modulation of NF-κB signaling, PEA + luteolin synergy	Paterniti et al. (56)
Luteolin	Tg2576 mice with moderate TBI	Aβ deposition ↓, GSK-3 activation ↓, phospho-tau ↓, pro-inflammatory cytokines ↓	Reduction of TBI-induced AD pathologies, anti-amyloid, anti-tau, anti-inflammatory effects	Sawmiller et al. (39)

AD, AD; ARCI, AD-related cognitive impairment; Lut, Luteolin; Exe, Exercise; CQ, Chloroquine; MWM, Morris water maze; GBE, Ginkgo biloba extract; NF-κB, Nuclear factor-kappa B; PI3K, Phosphoinositide 3-kinase; AKT, Protein kinase B; 3 × Tg-AD, Triple transgenic AD mice; PPARγ, Peroxisome proliferator-activated receptor gamma; Aβ, Amyloid beta; GFAP, Glial fibrillary acidic protein; Iba-1, Ionized calcium-binding adapter molecule 1; PEA, Palmitoylethanolamide; co-ultraPEALut, Co-ultramicronized palmitoylethanolamide/luteolin; OPCs, Oligodendrocyte precursor cells; PPAR-α, Peroxisome proliferator-activated receptor alpha; SAD, Sporadic AD; ICV-STZ, Intracerebroventricular streptozotocin; LUT-CHS, Luteolin-loaded chitosomes; DHA, Docosahexaenoic acid; UA, Urolithin A; PC-12, Pheochromocytoma cells; ER, Estrogen receptor; MAPK, Mitogen-activated protein kinase; L-Thea, L-Theanine; APS, Aversive phototaxis suppression; PCC, Protein carbonyl content; GST, Glutathione-S-transferase; GSH, Glutathione; LPO, Lipid peroxidation; AChE, Acetylcholinesterase; SOD, Superoxide dismutase; CAT, Catalase; BACE-1, β-site amyloid precursor protein cleaving enzyme; TBI, Traumatic brain injury; GSK-3, Glycogen synthase kinase-3.

Another work assessed the therapeutic efficacy of Ginkgo biloba leaf extract (GBE) in the context of AD (43). Significant constituents, including quercetin, luteolin, and kaempferol, were identified and predicted to exert their effects through the PI3K/AKT/NF-κB signaling pathway. In the murine model of AD, GBE was found to enhance cognitive performance, improve neuronal morphology, and diminish inflammatory markers, while also modulating pertinent protein and mRNA expression levels. These results indicated that GBE could potentially function as a promising therapeutic approach for AD due to its neuroprotective and anti-inflammatory properties (43).

He et al. (40) examined the neuroprotective properties of luteolin in triple transgenic models of AD (3 × Tg-AD) and in primary neuronal cultures. The administration of luteolin led to a marked enhancement in memory retention and cognitive capabilities by inhibiting the production of β-amyloid (Aβ), ameliorating mitochondrial dysfunction, and diminishing neuronal apoptosis. From a mechanistic standpoint, luteolin was found to directly activate peroxisome proliferator-activated receptor gamma (PPARγ), and the inhibition of PPARγ nullified its beneficial effects. These findings underscore the potential of luteolin as a therapeutic agent for AD through a PPARγ-mediated mechanism (40).

In addition, Tao et al. (44) assessed the synergistic effects of luteolin in conjunction with exercise on cognitive deficits in a mouse model of AD induced by Aβ1-42 oligomers. The combinatorial treatment yielded a significant enhancement in memory outcomes, surpassing the efficacy of each individual intervention, by mitigating Aβ accumulation, attenuating the activation of astrocytes and microglia, and promoting autophagy within the hippocampus and cortex. These results suggest that the combination of luteolin and exercise may represent a promising strategy to avert or postpone cognitive decline associated with AD (44).

An investigation was conducted to examine the impact of Aβ1-42 on the interactions between astrocytes and oligodendrocytes, while also assessing the neuroprotective efficacy of co-ultramicronized palmitoylethanolamide and luteolin (co-ultra PEALut) in an *in vitro* model simulating AD. The presence of Aβ1-42 resulted in astrocyte activation, inflammation, and hindered maturation of oligodendrocytes. Administration of co-ultra PEALut alleviated these detrimental effects, thereby maintaining oligodendrocyte structure and functionality, partially through the activation of PPAR-α. These results elucidated a novel mechanism for the restoration of oligodendrocyte homeostasis in AD (45). In another study, researchers formulated and assessed intranasal luteolin-loaded chitosan nanoparticles (LUT-CHS) aimed at addressing cognitive impairments in a sporadic AD (SAD) murine model. The LUT-CHS exhibited optimal physicochemical characteristics along with a high entrapment efficiency. *In vivo* analysis indicated that treatment led to improvements in both short-term and long-term memory, enhanced neuronal viability, a reduction in amyloid plaque burden, suppression of Aβ aggregation and hyperphosphorylated tau, as well as a decline in inflammation and oxidative stress. These findings propose that LUT-CHS represents a safe, effective, and non-invasive approach for alleviating Alzheimer's pathology and cognitive deficits (46).

Elsheikh et al. (47) assessed the efficacy of intranasal delivery of luteolin utilizing bile-salt-based nanobilosomes for the treatment of AD, thereby addressing the constraints imposed by the BBB. The optimized bilosomes exhibited desirable characteristics including particle size, zeta potential, entrapment efficiency, and prolonged drug release kinetics. *In vivo* analysis indicated that a 21-day treatment regimen significantly enhanced both short- and long-term memory, diminished oxidative stress and pro-inflammatory mediators, and inhibited the aggregation of amyloid-β as well as hyperphosphorylated Tau within the hippocampal region. The results substantiated that luteolin bilosomes represent a safe, non-invasive, and efficacious approach, providing superior cognitive enhancements compared to traditional luteolin suspension (47). Another rigorous investigation sought to identify a synergistic nutraceutical amalgamation as a prospective preventative intervention for AD. Docosahexaenoic acid, luteolin, and urolithin A were subjected to individual and combinatorial testing for their efficacy in mitigating Aβ1-42-induced cytotoxicity in human neuroblastoma cell lines. A triadic compound formulation exhibited the most pronounced protective effects. These findings indicate that this synergistic composition has the potential to serve as a foundational basis for the development of functional foods aimed at preventing or concurrently treating AD (48). In a conducted study by Ahmad et al. (49), it was examined the neuroprotective properties of luteolin in relation to Aβ1-42-induced neuroinflammation, amyloidogenesis, and synaptic impairment within a murine model of AD. Administration of luteolin (80 mg/kg/day) resulted in a significant reduction in the activation of p-JNK, p38 MAPK, GFAP, Iba-1, and various inflammatory markers such as p-NF-κB p65, tumor necrosis factor-alpha (TNF-α), interleukin-1 beta (IL-1β), a decrease in pro-apoptotic proteins (including Bax, Caspase-3, Cox-2), a downregulation of BACE-1 and Aβ1-42 concentrations, and an enhancement of synaptic markers (namely PSD-95, SNAP-25). Mechanistic investigations indicated that luteolin mediates these neuroprotective effects through the inhibition of JNK, underscoring its potential as a therapeutic agent for AD (49).

Kou et al. (50) determined the impact of luteolin on cognitive impairments and neuroinflammatory responses in 3 × Tg-AD murine models. Administration of luteolin (20–40 mg/kg/day for a duration of three weeks) resulted in a dose-dependent enhancement of spatial learning and memory capabilities, a reduction in astrocyte hyperactivation (GFAP), a suppression of pro-inflammatory markers (TNF-α, IL-1β, IL-6, NO, COX-2, iNOS), and a decrease in endoplasmic reticulum stress indicators (GRP78, IRE1α) within cerebral tissues. In an *in vitro* context, luteolin effectively inhibited lipopolysaccharide-induced glioma cell proliferation, inflammatory responses, and endoplasmic reticulum stress. These results elucidated that the neuroprotective attributes of luteolin are facilitated through the inhibition of endoplasmic reticulum stress in astrocytes, thereby endorsing its prospective application as a therapeutic agent for AD (50). Also, Ojo et al. (51) examined nine flavonoids derived from T. diversifolia, B. sapida, and I. gabonensis for their potential neuroprotective properties against AD targets, such as acetylcholinesterase (AChE), butyrylcholinesterase (BChE), and monoamine oxidase (MAO), employing pharmacophore modeling, AutoQSAR predictions, and molecular docking methodologies. Luteolin exhibited the most substantial binding affinity (-9.60 kcal/mol), succeeded by apigenin and ellagic acid, while all compounds displayed favorable absorption characteristics and non-toxic profiles. These findings indicated that these flavonoids, particularly luteolin, represent promising candidates for the advancement of pharmacological interventions targeting NDDs (51). A study investigated the protective effects of luteolin against Aβ25-35-induced apoptosis in PC-12 cells, serving as an *in vitro* model for AD. The results demonstrated that luteolin significantly enhanced cell viability, diminished apoptosis, upregulated Bcl-2 expression, and downregulated Bax and caspase-3 levels. Mechanistically, luteolin was found to activate the ER/ERK/MAPK signaling pathway through ERβ, while the application of inhibitors fulvestrant and U0126 partially mitigated these effects. These findings shown that luteolin may represent a promising therapeutic candidate for AD by inhibiting Aβ-induced neuronal apoptosis (52).

Park et al. (53) examined the synergistic effects of luteolin and l-theanine on symptoms analogous to AD in rats infused with Aβ(25–35). The combined therapeutic regimen (AD-LuTh) resulted in notable improvements in memory, augmented hippocampal insulin signaling through the pAkt → pGSK → pTau cascade, mitigated neuroinflammation, and rectified disturbances in glucose metabolism. Metabolomic analysis indicated a normalization of levels of proline, phenylpyruvic acid, normetanephrine, and norepinephrine. Collectively, luteolin and l-theanine exhibited a synergistic protective effect against cognitive deficits and metabolic dysfunction, thereby suggesting their potential utility as a therapeutic strategy to avert or postpone the progression of AD (53). Another study assessed the neuroprotective properties of luteolin utilizing a transgenic Drosophila model that expresses human Aβ42. Administration of dietary luteolin (5–20 μM) resulted in a dose-dependent enhancement of lifespan, sustenance of climbing proficiency, and amelioration of activity patterns. Biochemical evaluations indicated a reduction in lipid peroxidation, protein carbonyl content, AChE, SOD, CAT, and caspase 3/9 activities, concomitant with an elevation in GSH levels. Histopathological examinations demonstrated a decrease in Aβ42 deposition at elevated dosages. Molecular docking analyses proposed that luteolin interacts with AChE and Aβ42, thereby inhibiting enzymatic activity and obstructing plaque formation, thereby underscoring its potential therapeutic efficacy against AD-like pathologies (54).

The aim of a study was to determine the impact of luteolin on cognitive deficits in a streptozotocin (STZ)-induced rat model of AD. Behavioral evaluations, encompassing the Morris water maze and probe assessments, demonstrated that luteolin substantially enhanced spatial learning and ameliorated memory impairments induced by STZ. Histopathological examination indicated that luteolin inhibited the STZ-mediated diminution of CA1 pyramidal layer thickness. These observations suggested that luteolin confers protective benefits on both cognitive performance and hippocampal architecture in AD models (55). Another research effort assessed the neuroprotective properties of co-ultraPEALut, a synergistic formulation of palmitoylethanolamide (PEA) and luteolin, in both *in vitro* and *ex vivo* models of AD. Human SH-SY5Y neuronal cells and mouse hippocampal slices subjected to Aβ_1 − 42_ exhibited diminished cell viability, neuroinflammation, and apoptotic activity. Pre-treatment with co-ultraPEALut resulted in a reduction of glial activation, an enhancement of neuronal nitric oxide synthase and BDNF levels, and a decrease in apoptotic occurrences. These findings indicated that co-ultraPEALut effectively alleviates Aβ-induced neuroinflammation and neuronal injury (56). An investigation examined the ramifications of moderate traumatic brain injury (TBI) in Aβ-depositing Tg2576 murine models, elucidating the intersections between TBI and AD, which encompass augmented Aβ deposition, activation of GSK-3, phosphorylation of tau, and neuroinflammatory responses. Administration of the flavonoid luteolin markedly diminished these pathological alterations. These results imply that luteolin may function as a safe, natural intervention to avert or mitigate TBI-induced AD-like pathologies, thereby providing potential therapeutic advantages for individuals at heightened risk or those with a history of TBI exposure (39). Luteolin has been the subject of extensive research regarding its neuroprotective capabilities in models of AD. Through a variety of experimental approaches, including *in vitro, ex vivo*, and *in vivo* methodologies, luteolin, whether administered independently or in conjunction with adjunctive therapies such as physical exercise, Ginkgo biloba extract, L-theanine, DHA, urolithin A, or palmitoylethanolamide, has consistently exhibited enhancements in cognitive abilities, memory retention, and learning processes. At a mechanistic level, luteolin has been shown to exert its effects through multiple biochemical pathways, encompassing anti-inflammatory, antioxidant, anti-apoptotic, and anti-amyloidogenic mechanisms, as well as the modulation of autophagy, protection of mitochondrial function, promotion of neurogenesis, and regulation of insulin signaling pathways. The utilization of nanoparticle-based delivery systems, including chitosan-decorated nanoparticles and bilosomes, has further augmented the brain-targeting efficacy of luteolin, resulting in improved bioavailability and therapeutic outcomes. Collectively, these investigations underscore luteolin as a promising multi-target therapeutic candidate for the treatment of AD, with the potential to alleviate neuroinflammation, oxidative stress, amyloid and tau pathology, and neuronal dysfunction.

### 4.2 PD

PD represents a progressive neurodegenerative disorder characterized by the deterioration of dopaminergic neurons situated in the substantia nigra, in conjunction with the accumulation of α-synuclein aggregates, ultimately resulting in both motor and non-motor impairments (57). Current therapeutic strategies primarily provide symptomatic relief without halting the neurodegenerative trajectory, thus highlighting the critical need for pioneering disease-modifying interventions.

Consequently, the exploration of luteolin's role within the context of PD is of significant relevance.

An investigation systematically screened 98 flavonoids for their capacity to inhibit α-synuclein fibrillation, thereby identifying luteolin and baicalein as the most efficacious compounds (58). *In vitro* experimental assessments demonstrated that these flavonoids significantly curtailed α-synuclein fibril formation, diminished β-sheet content, and improved SH-SY5Y cell viability by transmuting toxic fibrils into non-toxic aggregates. These findings bolster the potential for flavonoid-based therapeutic interventions for synucleinopathies, such as PD (58) (Table 2). Dopamine is integral to optimal brain function, and its dysregulation is a key factor in the pathogenesis of PD.

**Table 2:** Summary of luteolin-based interventions and their mechanisms in Parkinson's disease models.

Type of intervention	Model	Main results	Mechanism of action	Reference
Luteolin & Baicalein against α-syn aggregation	*In silico, in vitro* (α-syn fibrillation assays; SH-SY5Y cells)	↓α-syn fibril formation; ↓β-sheet content; ↑ SH-SY5Y cell viability; ↓ toxic fibrils and ↑ non-toxic aggregates	Inhibition of α-syn aggregation; destabilization of toxic fibrils; conversion into non-toxic amorphous aggregates	Khorsand et al. (58)
Luteolin (25–50 mg/kg) in rotenone-induced PD	Wistar rats	↑ motor function; ↑ GSH; ↓ nitrate; ↓ TNF-α; ↓ Bax; ↑ dopamine; ↑ neuronal survival	Antioxidant effects (↑ GSH, ↓ nitrosative stress); anti-inflammatory effects (↓ TNF-α); anti-apoptotic effects (↓ Bax)	Chib et al. (59)
Bacopa monnieri compounds (quercetin, apigenin, luteolin)	*In silico, in vitro*	↑ binding affinity to α-syn, Aβ, tau; ↓α-syn production (quercetin); ↑ protection against rotenone toxicity (luteolin)	Inhibition of α-syn/Aβ/tau aggregation; antioxidant neuroprotection	Shukla et al. (60)
Luteolin (10–50 mg/kg) in 6-OHDA PD model—mood effects	6-OHDA rat model	↑ improvement in depression/anxiety behaviors; ↑ catalase; ↑ SOD; ↓ MDA; ↓ NF-κB, ↓ NLRP3, ↓ ASC, ↓ Caspase-1; ↓ IL-6, ↓ IL-1β, ↓ TNF-α; ↑ IL-10	Antioxidant effects (↑ CAT, ↑ SOD, ↓ MDA); anti-inflammatory effects through NF-κB/NLRP3 axis inhibition; ↑ IL-10	She et al. (61)
Luteolin in ER-stress 6-OHDA PD cell model	SH-SY5Y cells	↑ HRD1 and SEL1L expression; ↓ ER stress; ↓ cell death; – no protective effect when HRD1/SEL1L are silenced	Upregulation of ERAD pathway (HRD1, SEL1L); suppression of ER-stress-induced apoptosis	Nishiguchi et al. (63)
Palmitoylethanolamide/Luteolin (um-PEALut) as adjuvant therapy for camptocormia	Case report in a PD patient	↓ camptocormia severity; ↓ dyskinesia; ↑ responsiveness to levodopa; ↑ sustained improvement over 4 months; ↑ near-complete resolution of trunk and leg dyskinesia	Anti-inflammatory and neuroprotective effects of PEA + luteolin; likely enhancement of dopaminergic therapy responsiveness; modulation of neuroinflammation	Brotini et al. (65)
Humulus japonicus fraction enriched with luteolin-7-O-glucoside	Unilateral 6-OHDA-lesioned mice; *in vitro* MAO-B inhibition	↓ MAO-B activity (luteolin & apigenin strongest); ↑ motor behavior; ↓ motor asymmetry; ↓ ipsilateral rotations after 300 mg/kg HJF	MAO-B inhibition; neuroprotection through enhanced flavonoid content; antioxidant and anti-inflammatory pathways	Lee et al. (66)
Luteolin against rotenone toxicity in BV2 microglia	BV2 microglial cell model	↑ viability at low doses (1–5 μM); ↓ viability protection at high doses (25–50 μM); ↓ LDH release; ↓ IL-1β; – TNF-α unchanged; ↑ Nrf2; ↑ Trx1; ↑ Park2; ↓ Lrrk2; – Pink1 unchanged	Hormetic antioxidant response (low-dose benefit); regulation of PD-related genes (↑ Park2, ↓ Lrrk2); anti-inflammatory activity (↓ IL-1β); redox regulation via Nrf2/Trx1	Elmazoglu et al. (67)
Palmitoylethanolamide + Luteolin (co-ultraPEALut) in MPTP PD model	MPTP-induced PD mice and SH-SY5Y cells	↓ neuroinflammation; ↓ activated astrocytes; ↓ pro-inflammatory cytokines; ↓ iNOS; ↑ TH-positive neurons; ↑ autophagy markers (Beclin-1, p62); ↓ p70S6K; ↑ neuronal protection	Modulation of neuroinflammatory pathways; stimulation of autophagy (↑ Beclin-1, ↑ p62); inhibition of mTOR signaling (↓ p70S6K); neuroprotection through combined PEA + luteolin actions	Siracusa et al. (68)

HD, Huntington's disease; HTT, huntingtin protein; mHTT, mutant huntingtin; CAG, cytosine–adenine–guanine; polyQ, polyglutamine; ROS, reactive oxygen species; PD, Parkinson's disease; PEA, palmitoylethanolamide; um-PEALut, ultramicronized palmitoylethanolamide plus luteolin; MAO-B, monoamine oxidase B; HJF, Humulus japonicus fraction; 6-OHDA, 6-hydroxydopamine; IL-1β, interleukin-1 beta; TNF-α, tumor necrosis factor-alpha; Nrf2, nuclear factor erythroid 2–related factor 2; Trx1, thioredoxin-1; Park2, parkin gene; Lrrk2, leucine-rich repeat kinase 2; Pink1, PTEN-induced kinase 1; MPTP, 1-methyl-4-phenyl-1,2,3,6-tetrahydropyridine; TH, tyrosine hydroxylase; iNOS, inducible nitric oxide synthase; SH-SY5Y, human neuroblastoma cell line; p70S6K, ribosomal protein S6 kinase beta-1; Beclin-1, autophagy-related protein Beclin-1; p62, sequestosome-1.

Chib et al. (59) investigated the neuroprotective properties of luteolin within a rotenone-induced PD rat model. Administration of luteolin (25–50 mg/kg) resulted in enhanced motor performance, restoration of oxidative homeostasis via elevated glutathione levels and diminished nitrate levels, modulation of TNF-α and Bax to mitigate inflammation and apoptosis, and an increase in cerebral dopamine concentrations. Histopathological analysis corroborated the preservation of dopaminergic neurons, suggesting luteolin's potential as a therapeutic agent for PD through its antioxidant, anti-inflammatory, and anti-apoptotic properties (59).

Another study assessed the bioactive constituents of Bacopa monnieri against fundamental therapeutic targets, which include alpha-synuclein, amyloid beta, tau proteins, and serotonin receptors. Molecular docking and MM/GBSA analyses revealed Quercetin, Apigenin, and Luteolin as promising candidates for further exploration. *In vitro* investigations demonstrated that Quercetin effectively inhibited alpha-synuclein aggregation, whereas Luteolin exhibited significant preventive advantages. Additional *in vivo* research is warranted to substantiate their therapeutic and preventive efficacy in the contexts of PD and AD (60).

In a study by She et al. (61), it was sought to elucidate the antidepressant and anxiolytic properties of luteolin within a rat model of PD induced by 6-hydroxydopamine. Administration of luteolin at doses ranging from 10 to 50 mg/kg resulted in amelioration of behaviors indicative of depression and anxiety, enhancement of hippocampal antioxidant enzyme activity, attenuation of oxidative stress, and downregulation of NF-κB/NLRP3 inflammatory signaling pathways (61). Furthermore, luteolin treatment led to a reduction in pro-inflammatory cytokines concomitant with an elevation in interleukin-10 (IL-10) levels. Molecular docking studies corroborated the affinity of luteolin for the components of NF-κB/NLRP3, underscoring its potential as a neuroprotective agent in the context of mood disturbances associated with PD (61).

PD is associated with stress in the endoplasmic reticulum (ER), which subsequently activates the ubiquitin-proteasome system responsible for the degradation of aberrantly folded proteins. HRD1 and its stabilizing partner SEL1L are known to mitigate ER stress and neuronal apoptosis, thereby representing promising targets for therapeutic intervention (62). Another study has identified luteolin as a bioactive compound that enhances the expression of HRD1 and SEL1L within a cellular model of PD induced by 6-hydroxydopamine. Luteolin was observed to elevate the expression levels of these proteins, alleviate ER stress, and inhibit cellular apoptosis, suggesting its potential as a pioneering therapeutic agent for PD through mechanisms mediated by HRD1 and SEL1L (63). Camptocormia is prevalent in 3–18% of individuals diagnosed with PD and frequently exhibits inadequate responsiveness to levodopa treatment (64). A case report details the clinical presentation of a PD patient whose camptocormia manifested during “off” states and was managed using standard dopaminergic therapy in conjunction with a palmitoylethanolamide co-ultramicronized with luteolin (um-PEALut) (65). The introduction of um-PEALut resulted in an amelioration of dyskinesia, a reduction in camptocormia, and after a period of four months, culminated in the complete resolution of dyskinesia alongside a substantial decrease in camptocormia, underscoring its potential as an efficacious adjunct therapy for PD (65).

Humulus japonicus, historically utilized for a myriad of health conditions, was subjected to examination for its therapeutic efficacy in the context of PD. A total of fourteen distinct compounds, inclusive of a novel entity, were discerned from its fraction (HJF), wherein luteolin-7-O-glucoside and apigenin-7-O-glucoside emerged as principal constituents exhibiting notable enhancements of 12.57- and 9.68-fold, respectively, in comparison to ethanol extracts. Both apigenin and luteolin demonstrated potent inhibitory effects on monoamine oxidase B. In a 6-OHDA-induced PD murine model, the administration of oral HJF at a dosage of 300 mg/kg ameliorated motor asymmetry and deficits, thereby suggesting its promising potential to augment motor capabilities in PD via the action of bioactive flavonoid components (66). Rotenone elicits a pathology reminiscent of PD through the mediation of neuronal toxicity by microglial cells. The study evaluated the neuroprotective effects of luteolin in BV2 microglial cells subjected to rotenone exposure. Administration of low concentrations of luteolin (1–5 μM) resulted in enhanced cell viability, inhibition of lactate dehydrogenase (LDH) release, and a reduction in levels of IL-1β, concomitantly modulating the expression of genes pertinent to PD: specifically, an elevation in Park2, a reduction in Lrrk2, and an enhancement of Nrf2 and Trx1 expression. The levels of Pink1 and TNF-α remained unchanged. These results indicated that luteolin confers protective effects on microglia against oxidative stress and inflammation induced by rotenone, indicating its potential role as a hormetic neuroprotective agent in the context of PD (67).

Siracusa et al. (68) assessed a novel compound, co-ultraPEALut (a combination of palmitoylethanolamide and luteolin at a ratio of 10:1), in a murine model of PD induced by MPTP. Administration of this compound on a daily basis resulted in a reduction of PD-related biomarkers, activation of astrocytes, modulation of pro-inflammatory cytokines, and inhibition of inducible nitric oxide synthase. Furthermore, co-ultraPEALut was found to facilitate autophagic processes, as evidenced by an increase in Beclin-1 and p62 levels, alongside a decrease in p70S6K, findings that were corroborated in SH-SY5Y cell lines. These results indicated that co-ultraPEALut may confer neuroprotective effects in the context of PD by influencing neuroinflammatory responses and autophagic mechanisms (68).

Overall, these investigations elucidated the therapeutic potential of luteolin, palmitoylethanolamide/luteolin combinations, and compounds derived from natural products in the context of neurodegenerative diseases, particularly PD and HD. Throughout both clinical and preclinical models, luteolin uniformly exhibited neuroprotective, anti-inflammatory, antioxidant, and autophagy-modulating characteristics. Fractions extracted from plants, enriched with luteolin and apigenin from Humulus japonicus, ameliorated behavioral impairments in a 6-OHDA mouse model of PD, partially through the inhibition of MAO-B. In cultures of microglia, luteolin provided hormetic protection against the toxicity of rotenone by enhancing cellular viability at subtoxic doses, diminishing IL-1β release, alleviating oxidative stress markers, and modulating genes associated with PD, such as Park2 and Lrrk2. Collectively, these investigations emphasize the extensive neuroprotective capabilities of luteolin—mitigating neurotoxicity, attenuating inflammation, modulating oxidative stress responses, enhancing motor outcomes, and influencing the aggregation of pathological proteins. These converging findings indicate a promising therapeutic potential for luteolin and luteolin-based formulations in altering the disease mechanisms that underpin PD and HD.

Although AD and PD have distinct pathological hallmarks, several converging mechanisms contribute to their progression. In both disorders, neuroinflammation, oxidative stress, and mitochondrial dysfunction play central roles and are targets of luteolin's neuroprotective activity. However, luteolin's potential benefits in AD are primarily linked to the modulation of amyloid-β accumulation and tau hyperphosphorylation, whereas in PD its effects are more closely associated with the inhibition of α-synuclein aggregation, protection of dopaminergic neurons, and improvement of mitochondrial function, particularly at the level of complex I. These shared and disease-specific mechanisms highlight the multifaceted therapeutic potential of luteolin across NDDs.

### 4.3 Multiple sclerosis (MS)

MS represents a chronic autoimmune and neurodegenerative condition characterized by inflammation, demyelination, and progressive neurological deficits. Notwithstanding the advancements in immunomodulatory treatments, a substantial number of patients persist in experiencing disease progression, cognitive deterioration, and adverse effects related to therapy, thereby underscoring the necessity for safe complementary strategies (69). The integration of preclinical and preliminary clinical data emphasizes the importance of luteolin as a promising nutraceutical candidate for the modulation of immune responses, attenuation of neuroinflammation, and enhancement of neurological outcomes in multiple sclerosis.

An investigation unveiled 40 uniquely expressed genes linked to lipid metabolism (LMRGs) and underscored AKR1C3, NFKB1, and ABCA1 as pivotal markers for MS (70). Functional evaluations unveiled a rich tapestry of enrichment in arachidonic acid, steroid hormone pathways, fatty acid elongation, and the intricate world of sphingolipid metabolism. A sophisticated artificial neural network (ANN) model adeptly forecasted MS with impressive accuracy (AUCs 0.822–0.890). Immune cell exploration and ceRNA networks shed light on the underlying disease mechanisms, while molecular docking proposed luteolin, isoflavone, and thalidomide as promising therapeutic contenders (70) (Table 3). The exploration delved into the effects of luteolin, both in isolation and in partnership with interferon-beta (IFN-β), on peripheral blood mononuclear cells (PBMCs) derived from individuals battling MS. Luteolin exhibited a dose-dependent ability to curtail PBMC proliferation, diminish the pro-inflammatory cytokines IL-1β and TNF-α, and reduce the MMP-9/TIMP-1 ratio by inhibiting MMP-9 synthesis. In conjunction with IFN-β, luteolin showcased synergistic effects in regulating cell proliferation, cytokine secretion, and the MMP-9/TIMP-1 equilibrium, underscoring its promise as an immunomodulatory potential in the realm of MS (71).

**Table 3:** Summary of luteolin-based interventions and their mechanisms in multiple sclerosis and Huntington's disease models.

Type of intervention	Model	Main results	Mechanism of action	Reference
Identification of lipid metabolism-related gene markers and construction of a diagnostic model for MS	Bioinformatics analysis of gene expression profiles from MS patients	↑ AKR1C3, ↑ NFKB1, ↑ ABCA1; ANN model AUC: 0.826 (training), 0.822–0.890 (test sets); ↑ immune cell infiltration (Gamma.delta.T.cell, NK T.cell, Plasmacytoid DC, Regulatory T.cell, Type 1 T.helper.cell)	Dysregulated lipid metabolism; hub genes linked to MS pathogenesis; luteolin, isoflavone, and thalidomide predicted to bind hub genes and modulate signaling	Yang et al. (70)
Immunomodulatory effects of luteolin with or without IFN-beta on PBMCs from MS patients	*In vitro* PBMC cultures	↓ PBMC proliferation; ↓ IL-1β and TNF-α; ↓ MMP-9/TIMP-1 ratio; additive effects with IFN-beta	Anti-inflammatory and immunomodulatory; luteolin inhibits pro-inflammatory cytokine release and regulates extracellular matrix remodeling	Sternberg et al. (71)
Luteolin treatment	N171-82Q transgenic and WT mice	↑ Survival, ↑ Body weight maintenance, ↑ Motor coordination and balance, ↓ Motor dysfunction, ↓ Serum NfL, ↓ Huntingtin aggregates	Neuroprotective; reduces huntingtin aggregation and neurodegeneration	Mohammed et al. (73)
3-alkyl luteolin derivatives (Lut-C4, Lut-C6)	Striatal cells from HD knock-in mice (STHdh(Q111/Q111))	↓ Caspase-3 activity, ↓ ROS, ↑ Cell viability, ↑ Nrf2 nuclear levels, ↑ SOD1 and GCLc expression	Antioxidant; activates Nrf2/ARE transcriptional pathway, enhances cellular antioxidant defenses	Oliveira et al. (74)
Luteolin dietary supplementation	Transgenic Drosophila HD model	Dose-dependent ↑ climbing ability, ↓ Oxidative stress	Antioxidant; reduces oxidative stress, positive interaction with mutant huntingtin (mHTT)	Siddique et al. (75)

LMRGs, Lipid Metabolism-Related Genes; ANN, Artificial Neural Network; PBMCs, Peripheral Blood Mononuclear Cells; IL-1β, Interleukin-1 beta; TNF-α, Tumor Necrosis Factor alpha; MMP-9, Matrix Metalloproteinase-9; TIMP-1, Tissue Inhibitor of Metalloproteinases-1; IFN-β, Interferon-beta; HD, Huntington's Disease; NfL, Neurofilament light chain; ROS, Reactive Oxygen Species; Nrf2, Nuclear factor erythroid 2, related factor 2; SOD1, Superoxide Dismutase 1; GCLc, Glutamate-Cysteine Ligase Catalytic subunit; mHTT, Mutant Huntingtin protein.

### 4.4 Huntington's disease (HD)

HD constitutes a genetic neurodegenerative condition distinguished by the aggregation of mutant huntingtin proteins, a progressive decline in striatal and cortical neuronal populations, motor impairments, psychiatric manifestations, and cognitive deterioration (72). Present therapeutic approaches are predominantly symptomatic in nature and fail to halt the process of neurodegeneration, thereby underscoring an urgent imperative for disease-modifying strategies that target protein aggregation, oxidative stress, and neuroinflammatory processes.

Preclinical investigations provide support for the exploration of luteolin within Huntington's disease models, given the compound's multifaceted actions. Collectively, the preclinical profile of luteolin suggests its potential as a promising nutraceutical agent for Huntington's disease, warranting dedicated translational research aimed at evaluating its capacity to attenuate neurodegeneration and safeguard motor and cognitive capabilities.

An investigation assessed the efficacy of luteolin in the context of HD utilizing N171-82Q transgenic murine models (73). Administration of luteolin was associated with enhanced survival rates, prevention of weight loss, and improvement in motor coordination and balance capabilities. Furthermore, it resulted in a significant reduction of serum neurofilament light chain (NfL) levels and a decrease in the accumulation of huntingtin aggregates within the cortical, hippocampal, and striatal regions. These results exhibited that luteolin may represent a viable therapeutic candidate for HD, demonstrating the potential to improve survival, alleviate motor impairments, and diminish the aggregation of pathogenic proteins, thereby addressing the existing deficiency in effective interventions for the progression of this debilitating condition (73).

Another research scrutinized the effects of luteolin (Lut) alongside four of its derivatives (Lut-C1, C4, C6, C10) on striatal cells afflicted by HD [STHdh(Q111/Q111)] (74). Both Lut-C4 and Lut-C6 markedly diminished caspase-3 activity and the levels of ROS, thereby promoting cell survival. They stimulated the activation of nuclear phospho-Nrf2 and enhanced the transcriptional activity of Nrf2/ARE, with Lut-C6 elevating the expression and function of SOD1 and GCLc, while Lut-C4 triggered GCLc mRNA expression. These discoveries underscore Lut-6′s potential as a powerful antioxidant candidate for therapeutic interventions in the realm of HD (74).

**Figure 2:**
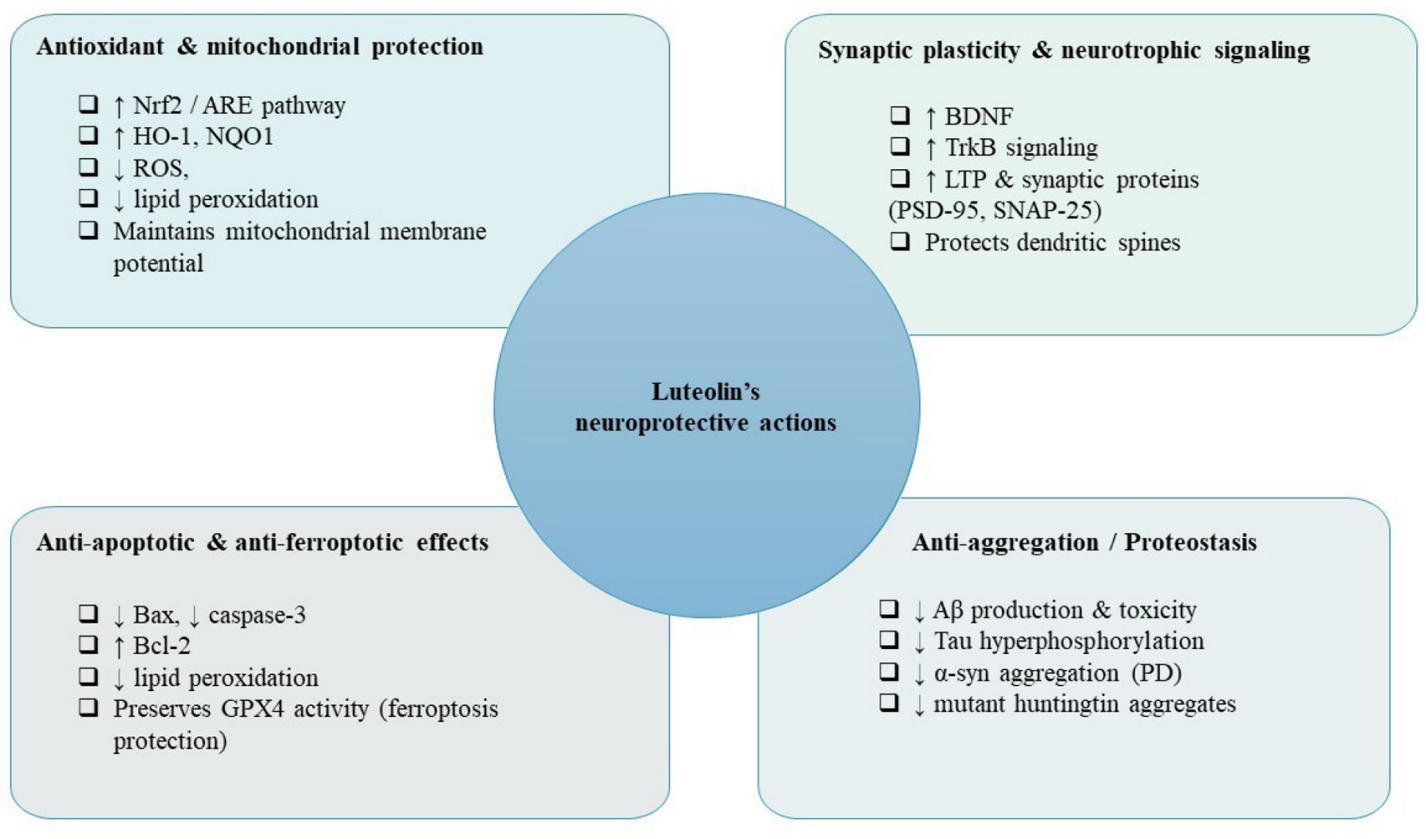
Core neuroprotective mechanisms of luteolin within the brain. The figure summarizes luteolin's principal intracellular actions, including activation of antioxidant defenses, enhancement of synaptic and neurotrophic signaling, inhibition of apoptotic and ferroptotic pathways, and reduction of pathogenic protein aggregation. These mechanisms represent the fundamental molecular pathways through which luteolin exerts neuroprotection.

Another investigation assessed the efficacy of luteolin in a transgenic Drosophila model of HD. Administration of dietary luteolin at concentrations ranging from 25 to 100 μM demonstrated a dose-dependent attenuation of the decline in climbing ability and a decrease in oxidative stress levels. Molecular docking studies revealed a favorable interaction between luteolin and mHTT. These data proposed that luteolin as a promising therapeutic candidate for alleviating symptoms associated with HD through its antioxidant and neuroprotective properties (75) (Figure 2).

## 5 Challenges, limitations, and future perspectives

The challenges, constraints, and prospective avenues pertaining to the therapeutic application of luteolin underscore essential factors for its effective conversion into clinical practice. Notwithstanding the robust preclinical data, luteolin is impeded by suboptimal bioavailability attributed to inadequate solubility, swift metabolic degradation, and insufficient stability, thereby necessitating sophisticated delivery methodologies such as nanocarriers, liposomal formulations, polymeric nanoparticles, and phospholipid complexes to augment its absorption and penetration into the central nervous system. A further significant constraint resides in the translational divide between animal models and human NDDs; existing models inadequately emulate the chronic, multifactorial, and age-related characteristics of ailments such as AD, PD, multiple sclerosis, and Huntington's disease. This divergence complicates the forecasting of human reactions and emphasizes the necessity for more advanced disease modeling techniques. Furthermore, the ideal dosage, administration frequency, and long-term safety profile of luteolin are yet to be adequately characterized, given the scarcity of clinical investigations aimed at evaluating potential adverse effects or interactions with standard therapeutic interventions. Subsequent studies ought to prioritize multi-omics methodologies encompassing genomics, proteomics, metabolomics, and epigenomics, to clarify individualized responses and underlying mechanistic pathways. Large-scale, rigorously controlled clinical trials are imperative to authenticate therapeutic efficacy, while the exploration of synergistic combination strategies with various nutraceuticals, anti-inflammatory agents, and conventional pharmacological treatments may further augment luteolin's neuroprotective capabilities.

## 6 Conclusions

The expanding corpus of empirical evidence underscores luteolin as a potentially effective neuroprotective nutraceutical, exhibiting multimodal mechanisms that specifically address the fundamental pathological processes associated with NDDs. Its mechanisms of action encompass the robust attenuation of neuroinflammation through the modulation of NF-κB, MAPK, and pathways associated with microglial activation; the augmentation of antioxidant defenses mediated by Nrf2 signaling and mitochondrial safeguarding; the facilitation of synaptic plasticity via mechanisms linked to BDNF; and the reduction of toxic protein aggregation involving Aβ, tau, α-synuclein, and mutant huntingtin. Collectively, these mechanistic elucidations reinforce luteolin's potential to preserve neuronal integrity and foster cognitive resilience. In summary, the preclinical findings consistently validate luteolin's efficacy in enhancing memory, motor skills, demyelination, and neuronal viability across various models of AD, PD, multiple sclerosis, and Huntington's disease. Preliminary clinical observations, albeit limited in scope, provide additional support for its prospective advantages in diminishing inflammation and augmenting cognitive function, particularly within the contexts of AD and multiple sclerosis. Looking ahead, luteolin emerges as a compelling candidate for integrative methodologies aimed at decelerating neurodegeneration and alleviating cognitive decline. Subsequent endeavors ought to concentrate on the optimization of delivery mechanisms, the execution of extensive clinical trials, and the investigation of personalized modalities alongside combination therapies to fully exploit its therapeutic efficacy and translate these advantages into viable clinical applications.
